# Post-Transcriptional Gene Regulation by HPV 16E6 and Its Host Protein Partners

**DOI:** 10.3390/v14071483

**Published:** 2022-07-06

**Authors:** Caylin L. Billingsley, Sreenivasulu Chintala, Rachel A. Katzenellenbogen

**Affiliations:** 1 Herman B Wells Center for Pediatric Research, Department of Pediatrics, Indiana University School of Medicine, Indianapolis, IN 46202, USA; caybilli@iu.edu (C.L.B.); srchinta@iu.edu (S.C.); 2 Department of Microbiology and Immunology, Indiana University School of Medicine, Indianapolis, IN 46202, USA

**Keywords:** human papillomavirus, E6, NFX1-123, PABPCs, RNA regulation, cervical cancer, hTERT

## Abstract

Human papillomavirus type 16 (HPV 16) is the most common oncogenic type of HPV in cervical, anogenital, and head and neck cancers, making HPV 16 an important high-risk HPV (HR HPV) type. To create an environment permissible for viral maintenance and growth and to initiate and support oncogenesis, the HR HPV protein E6 functions to dysregulate normal cellular processes. HR HPV type 16 E6 (16E6) has previously been shown to bind cellular proteins in order to transcriptionally activate genes and to target regulatory proteins for degradation. We have identified an additional functional model for 16E6. First, 16E6 binds to cellular RNA processing and binding proteins, specifically cytoplasmic poly(A) binding proteins (PABPCs) and NFX1-123. Then, 16E6 hijacks those proteins’ functions to post-transcriptionally regulate cellular immortalization, growth, and differentiation genes and pathways in keratinocytes. In this review, we have highlighted studies that introduce this new model of 16E6 functionality. Understanding ways in which HR HPV dysregulates cellular processes—particularly at the level of post-transcriptional gene regulation—presents new ways to consider mechanisms underlying DNA tumor virus function and new areas for therapeutic target development in HPV-associated cancers.

## 1. Introduction

Human papillomaviruses (HPVs) are small, nonenveloped, double-stranded DNA viruses that are part of the Papillomaviridae family. There are over 200 types of HPVs currently enumerated, with nearly 400 types of papillomaviruses identified to date. HPV types differ based on the DNA sequence of their L1 gene; differences greater than 10% in a sequence define a unique—and new—HPV type [1]. HPV types are phylogenetically clustered into genera and species, and that also aligns with their ability to infect cutaneous or mucosal epithelium [2]. Among all HPV types, there is a wide range of clinical manifestations from an infection; these infection differences are primarily based on the HPV type. It is known that high-risk (HR) HPVs, which cluster in species 7 and 9 of the alpha papillomavirus genus [1,2], are the causative agents of nearly all cervical cancers, and they have been implicated in a significant percentage of oropharyngeal and anogenital cancers [3,4]; however, the mechanisms underlying many components of an HR HPV infection, as well as its advancement from infection to cellular dysplasia, and ultimately to cancer, are not well investigated.

First, to understand the manner by which HPVs establish infections, whether HR or low risk (LR) for cancer, we need to study the common functions of all HPV genes. Then, to determine the manner by which HR HPVs initiate and drive cancer development and progression after infection, we must parse the roles of HR HPV genes that function as oncogenes—required for malignant cellular pathway activation and maintenance in the host cell. Furthermore, within HR HPV types, there are common and unique gene expressions and pathway activations that merit detailed evaluation.

Focusing on the commonalities of HPVs, all HPVs are double-stranded DNA viruses whose genomes are polycistronic. HPV genes are expressed using cellular polymerases and RNA processing proteins in order to produce both full-length gene transcripts, such as HPV E6 and E7, as well as splice variants of genes, such as HPV E6*, E8^E2, and E1^E4. Seminal studies of RNA splicing factors as well as cytoplasmic poly(A) binding proteins have been conducted that define the critical role post-transcriptional gene regulation has in HPV gene expression and function during the HPV life cycle [see review article, 5].

Our laboratory studies HR HPVs, and we are interested in elucidating the way HR HPV viral oncogenes E6 and E7 dysregulate and co-opt host cell factors to not only complete the HPV life cycle and support a persistent infection but also to initiate and maintain oncogenesis in its infected host cells. We have conducted nearly all of our studies with HPV type 16 (HPV 16) as our model for HR HPV. This is because HPV 16 is the most common oncogenic type found in cervical cancer, as well as anogenital and head and neck cancers, making HPV 16 a critical HR HPV type to study. In our work, we have identified that HPV 16 E6 (16E6) hijacks host cell RNA processing and binding proteins, specifically cytoplasmic poly(A) binding proteins (PABPCs) and NFX1-123, to drive cellular immortalization and to tip the balance of cellular growth and differentiation within keratinocytes. These pathways (immortalization, growth, and differentiation) are commonly modulated during HR HPV infections and during HPV-associated cancers. Furthermore, HPV gene expression itself normally is controlled by RNA splicing and by transcriptional stabilization [5]. However, the post-transcriptional gene regulation of cellular targets directed by 16E6 is novel and represents a new model of HPV gene and pathway dysregulation. It is notable that this is not a new concept for other viruses. Several studies have demonstrated that both RNA and DNA viruses have been shown to dysregulate cellular RNA stability and translation during viral infections to create an environment suitable for the virus [6,7,8]; however, very little evidence of this has been shown in HPV.

Below, we will describe: the known functions of 16E6; the known functions of its protein partner, NFX1-123, which also binds cytoplasmic poly(A) binding proteins (PABPCs); and the studies we and others have conducted that support an additional form of virus-driven host gene regulation—post-transcriptional binding and stabilization of host mRNA that affects cellular function and longevity in culture. Additionally, we will highlight the potential clinical significance of this model in HPV-associated cancer development as well as future studies that will broaden our understanding of this form of gene and pathway regulation during HPV-associated cancer development and progression.

## 2. Functions of 16E6

E6 and E7 are two viral proteins that are responsible for creating a permissive cellular environment for viral replication [9]. Keratinocytes in the basal layer of stratified squamous epithelium normally are the only cells that can grow, replicate their DNA, and divide. Once in the suprabasal and upper layers of stratified squamous epithelium, typically, keratinocytes begin to differentiate and no longer divide. E6 and E7 expression support continued host cell DNA replication, cellular growth, and cellular division in these upper layers of the stratified squamous epithelium [2]. It is important to note that splice variants of the E6 gene (E6* isoforms) are expressed during infection. Previous studies have characterized the E6* transcripts as primarily functioning to facilitate the translation of the E7 gene [10,11], and despite HPV16 E6*I being the most abundantly expressed E6* isoform, it does not lead to increased keratinocyte immortalization or proliferation [10]. With this in mind, this review will focus on studies of the full-length E6 protein.

**Figure 1:**
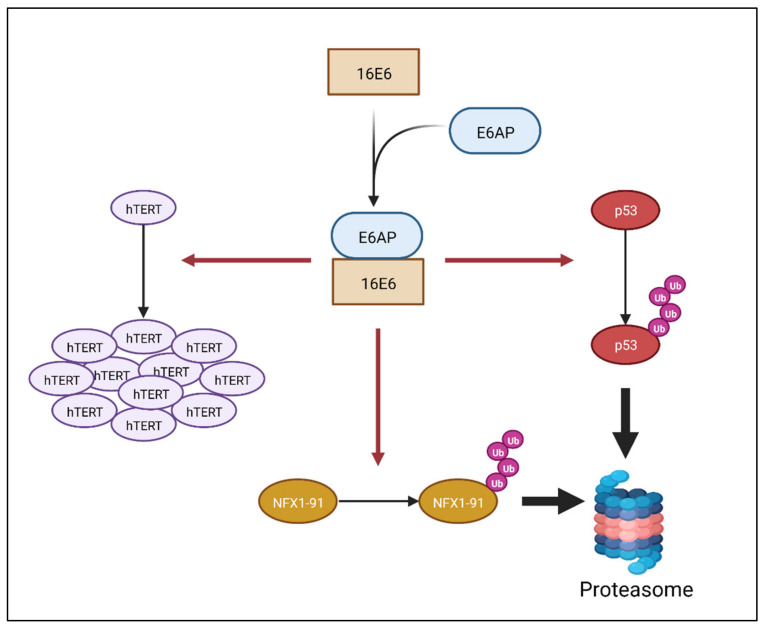
**Some of the known functions of HPV 16E6**. HPV 16E6 partners with the cellular protein E6AP, which enables the oncoprotein to polyubiquinate cellular proteins, such as p53 and NFX1-91. In partnership with E6AP, 16E6 has also been shown to increase telomerase activity by increasing the expression of hTERT, the catalytic and rate-limiting subunit of telomerase. Figure created with Biorender.com.

In HR HPV infections, the E6 protein itself has no enzymatic function. E6 is only able to direct effects in its host cell through protein partnerships with cellular factors. One key partner for HR HPV E6 (HR E6) is the E3 ubiquitin ligase E6-associated protein (E6AP). HR E6 and E6AP have been shown to polyubiquitinate p53 and target it for proteasomal degradation (Figure 1) [12,13]. This degradation of p53 permits dysregulated growth of keratinocytes infected by HPV. It blocks cellular apoptosis and allows for more efficient replication of the viral genome during the maintenance and amplification stages of the HPV life cycle.

HR E6 with E6AP have also been found to activate telomerase in cells (Figure 1), and this telomerase activity increases further as the infection is maintained [14]. All HPV-associated cancers have telomerase activity, and when this is reduced, cells no longer grow in culture. Telomerase is an enzyme that extends the repetitive sequences of telomeric DNA at the ends of linear chromosomes. Telomerase is normally active in stem cells; however, in normal somatic diploid cells, telomerase is not active and telomeric DNA shortens with each cellular division, marking the age of a cell. After 40 to 60 DNA replications and cellular divisions, telomeric DNA becomes critically shortened (named the Hayflick limit), and cells undergo either senescence or apoptosis [15]. However, if telomerase is active, telomeric DNA is extended during DNA replication; this allows cells to avoid senescence as cellular division occurs, and this is required for cellular immortalization.

Telomerase is a ribonucleoprotein, and human telomerase reverse transcriptase (hTERT) is the catalytic subunit of the telomerase enzyme in humans. Its expression is rate-determining in cells, and hTERT is normally constitutively repressed in somatic, diploid cells. This repression occurs at the hTERT promoter, with transcriptional repressors bound to the promoter’s *cis*-elements. Previous studies have demonstrated that 16E6 and E6AP transcriptionally derepress, or activate, hTERT at its promoter through some of the following mechanisms: removal of transcriptional repressors, such as NFX1-91 (Figure 1) [16,17,18,19]; interactions with transcriptional activators, such as c-Myc; and by binding to the promoter themselves. Mutational studies of the E6 oncoprotein have identified unique amino acids in 16E6 that regulate hTERT activation at the promoter and are separate from its role in degrading the p53 protein [20]. Therefore, 16E6 has two key roles in oncogenesis—avoiding apoptosis and inducing cellular immortalization. One regulation occurs at the level of protein through targeted degradation, and the other occurs at the level of DNA through transcriptional activation.

There are additional roles that HR E6 has in the HPV life cycle and in oncogenesis through promoter regulation and protein degradation. Those are described elsewhere and are summarized in Figure 1. Interestingly, we and others have identified that 16E6 supports the HPV life cycle and oncogenesis at a third level—that of RNA. This occurs through host cell protein partnerships outside E6AP; they include NFX1-123 and PABPCs. It has been demonstrated that the E6 protein has different binding domains that interact with its cellular binding partners. 16E6 directly binds to E6AP by way of the LXXLL motif [21], and HR E6 proteins bind to PDZ motif-containing proteins through its terminal four amino acids [22,23,24,25]. However, to date, the motif of 16E6 that binds either NFX1 isoform has not been defined.

## 3. 16E6 Interaction with NFX1-123

**Figure 2:**
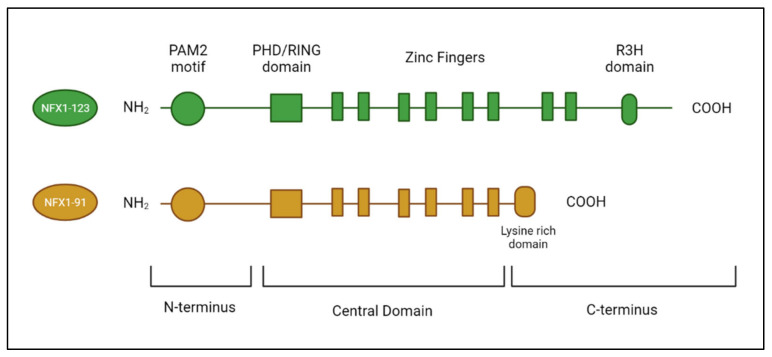
**Splice variants of NFX1**. The two splice variants of NFX1 (NFX1-123 and NFX1-91) share a common N-terminus, which contains a PAM2 motif, and a central domain, which contains a PHD/RING and six zinc-like fingers. The C-terminus of NFX1-123 contains two additional zinc-like fingers and an R3H domain. The C-terminus of NFX1-91 is truncated and lysine-rich. Figure created with Biorender.com.

**Figure 3:**
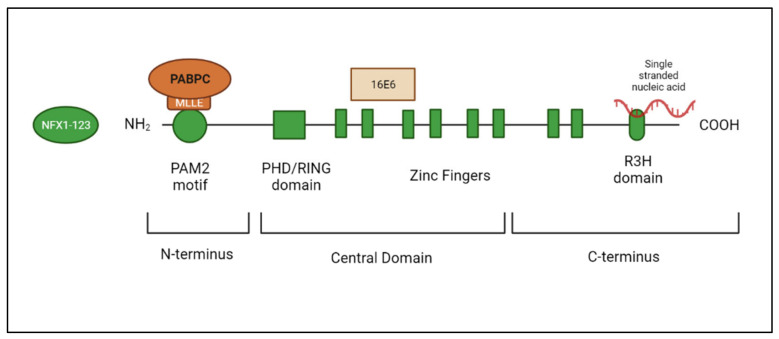
**Binding partners of NFX1-123**. The MLLE domain of PABPCs binds to NFX1-123 at its N-terminal PAM2 motif. HPV 16E6 binds within the central domain. The R3H domain of the C-terminus has putative single-stranded nucleic acid binding capabilities. Figure created with Biorender.com.

Nuclear X-box Factor 1 (NFX1) was initially discovered in the early 1990s in a screen for proteins that interacted with the X-box region of the Class II major histocompatibility genes [26]. Further examination of the *NFX1* gene in eukaryotic cells identified two splice variants that produce two different protein isoforms (Figure 2). The shorter splice variant, NFX1-91, is a 91 kDa protein, while the longer splice variant, NFX1-123, is a 123 kDa protein [16]. These isoforms share a common N-terminus and central domain but have unique C-termini. In the N-terminus, there is a PAM2 motif [27]. PAM2 motifs are found in proteins that directly bind PABPCs at their PABC (also known as MLLE) domain [28]. In the central domain, NFX1-91 and NFX1-123 both have a PHD/RING domain and six zinc-like fingers. The PHD/RING domain has E3 ubiquitin ligase functionality. The zinc-like fingers support direct DNA binding. NFX1-91 has a short, unique C-terminus that is 25 amino acids long. It is lysine-rich and is required for the NFX1-91 protein to be targeted for ubiquitin-mediated degradation, shortening its half-life relative to NFX1-123 [16]. The NFX1-123 unique C-terminus includes two additional zinc-like fingers and an R3H domain with putative single-stranded nucleic acid binding capabilities (Figure 3). The NFX1-91 protein is primarily located in the nucleus of cells, where it binds to gene promoters at X-boxes to regulate transcriptional activation or repression. In contrast, the NFX1-123 protein is primarily located in the cytoplasm of cells [29].

A yeast two-hybrid screen to identify novel protein partners with 16E6 and E6AP revealed, and co-immunoprecipitation assays confirmed, that 16E6 binds to both NFX1 protein isoforms (binding location noted in Figure 3) [16,27]. The 16E6/E6AP complex was identified not only bound to NFX1-91 and NFX1-123 but that NFX1-91 was targeted for polyubiquitination and degradation by 16E6/E6AP at its unique C-terminus [16]. NFX1-123 also was bound by 16E6, but it was not polyubiquitinated or degraded by 16E6 [16]. In fact, in a tandem affinity purification assay, NFX1-123 bound USP9X (also known as FAF-X), a ubiquitin-specific protease that can remove ubiquitin moieties from proteins [27]. More recent studies have also identified that NFX1-123 bound USP9X, and in the presence of 16E6, USP9X expression was increased [30]. This increase led to the deubiquitination of NFX1-123 and further stabilized the NFX1-123 protein [30].

These studies collectively identified new protein partners for HR E6, specifically 16E6. Not only did 16E6 bind *NFX1* gene products, but we have determined that NFX1-123 expression is increased in cervical dysplasia samples and is highly expressed in cervical cancers and cervical cancer cell lines [31]. Additionally, in The Cancer Genome Atlas cervical cancer data set, 13.2% of genes correlated with *NFX1* expression; among those correlative genes, there was significant enrichment for those associated with protein binding and RNA binding [32]. These data emphasize the value of understanding how 16E6 may increase the expression and co-opt the typical function of NFX1-123 and, based on the protein motifs found in NFX1-123, how 16E6 may utilize this host protein to drive oncogenesis in keratinocytes.

## 4. NFX1-123 and PABPCs

As noted above, in its N-terminus, NFX1-123 contains a cytoplasmic poly(A) binding protein interacting motif, PAM2, which is required to bind to PABPCs at their C-terminus [33] MLLE motif (Figure 3) [27,28]. PABPCs bind to messenger RNAs that have been spliced, capped, and poly(A) tailed, helping to shuttle them from the nucleus to the cytoplasm [34,35]. They bind to the poly(A) tail of mRNAs through their C-terminus RNA recognition motifs (RRMs), and they bind other proteins typically found at the mRNA cap through their C-terminus MLLE motif [33]. These protein–RNA interactions create a closed-loop structure for the mRNA [36]. It helps both stabilize the transcription and recruit and maintain translational machinery on the mRNA to increase protein production [34].

There are several PABPCs. PABPC1 binds poly(A) sequences and is expressed in three-fold excess to the number of poly(A) binding sites on all mRNA [34,36]. PABPC4 binds both poly(A) and poly(AU) sequences, where mRNA with a poly(AU) is less stable [37]; PABPC4 is also highly expressed in all cells but 10–15-fold less than PABPC1 [38]. PABPC1 and PABPC4 do not have entirely redundant functions or mRNAs to which they bind. Studies of knocked down embryonic PABP, PABPC1, or PABPC4 during vertebrate development identified unique developmental defects that could not be rescued fully by a different PABPC [39]. We confirmed that NFX1-123 binds both PABPC1 and PABPC4 at its PAM2 motif, and its interaction with PABPC4 appears to be high affinity (Figure 3) [27,29]. Because we found NFX1-123 to be a cytoplasmic protein, it had both a PAM2 motif and an R3H domain, and it bound to RNA processing proteins, such as PABPC1 and PABPC4, we wanted to determine whether NFX1-123, PABPCs, and 16E6 regulated gene expression post-transcriptionally. We focused on PABPC4 for three reasons: PABPC4 bound NFX1-123 at its PAM2 motif with high fidelity; it could be further overexpressed in cells, as its endogenous expression was not overly abundant; and we could knock down PABPC4 expression specifically or broadly with all PABPCs using targeted short hairpin RNAs.

## 5. 16E6, NFX1-123, and PABPC4 Synergistically Interact to Augment hTERT mRNA Expression

Seminal studies have shown that 16E6 activates hTERT expression and telomerase activity in keratinocytes [20,40]. It does so in two ways. First, it binds to E-box cis-elements in the hTERT promoter to transcriptionally activate the promoter [14]. Second, NFX1-91 is constitutively bound to the hTERT promoter at an X-box cis-element that overlaps with an E-box in the promoter downstream of the transcriptional start site but upstream of the translational start site [16,18]. When bound to the hTERT promoter, NFX1-91 constitutively represses hTERT transcription [18]. With 16E6 expression, 16E6 and E6AP bind to NFX1-91 and polyubiquitinate it at its unique C-terminus. This targets NFX1-91 for ubiquitin-mediated degradation to derepress the hTERT promoter [18,41], permitting transcription and translation of hTERT.

We and others have examined the interactions between 16E6, NFX1-123, and PABPC4 in relation to hTERT expression [27]. Previous ChIP assay studies in the Galloway laboratory confirmed that, unlike NFX1-91, NFX1-123 did not bind to the X-box sequence in the minimal core promoter of hTERT in keratinocytes [16], and NFX1-123 is not found in the nucleus of keratinocytes [29]. However, as described below, when the promoter of hTERT was cloned into a luciferase reporter construct, greater expression of NFX1-123 and PABPC4 with 16E6 did increase luciferase activity. Of note, this minimal core hTERT promoter region contains 710 nucleotides upstream of the hTERT transcription start site as well as the 5′ UTR region of hTERT, which has E-box and X-box cis-binding sites [27]. The 5′ UTR sequence is transcribed when the hTERT promoter is activated and fused to the luciferase mRNA produced from the reporter construct. 

In keratinocytes, transfection of this hTERT minimal core promoter-luciferase construct with an empty vector control led to no luciferase activity [27]. However, when 16E6 was also transfected, there was an increase in the luciferase activity above the vector control [27]. By itself, transfection of NFX1-123 with the luciferase construct did not increase luciferase activity; yet, when 16E6 and NFX1-123 were both transfected with the luciferase construct, there was an even greater increase in the luciferase activity [27]. This indicated that, in the presence of 16E6, NFX1-123 had the ability to augment the luciferase activity in this assay. Finally, when 16E6, NFX1-123, and PABPC4 were all transfected in concert with the luciferase construct, there was a 10-fold increase in the luciferase activity compared with 16E6 and NFX1-123 [27]. Therefore, 16E6 was required to activate the hTERT core promoter in this study, NFX1-123 could further increase expression from the reporter construct once activated, and PABPC4 synergistically augmented this luciferase activity. This highlighted the collaborative effect NFX1-123 and PABPC4 had with 16E6 in hTERT promoter-driven expression. Interestingly, it also demonstrated the potential role of hTERT post-transcriptional regulation by these three proteins. This is because the NFX1-123 protein is not typically found in the nucleus [29] or bound directly to the hTERT promoter [16]. However, if NFX1-123 was affecting the mRNA of hTERT, the mRNA produced by this luciferase reporter construct would include the 5′ UTR of hTERT. That would then give the luciferase mRNA sequence gene specificity that could be sensed, bound, or regulated by NFX1-123, which could, in turn, recruit PABPC4 to the mRNA [27].

Indeed, in these luciferase assays, the transfection of PABPC4 with 16E6 did not drive an increase in the luciferase activity compared with 16E6 addition alone, implying that PABPC4 was brought to the 5′ UTR hTERT/luciferase fused mRNA by NFX1-123 [27]. To study this with more granularity, we mutated and deleted the PAM2 motif in NFX1-123 to determine its impact on luciferase activity from this reporter construct. When the mutated or deleted PAM2 NFX1-123 constructs were transfected with 16E6 and PABPC4, the high activity of luciferase observed with wild-type NFX1-123 was significantly lowered [27]. This demonstrated the importance of recruiting PABPC4 by NFX1-123 for its function with 16E6 and, more broadly, the importance of RNA binding and processing proteins to the maximal expression of hTERT in keratinocytes [27].

This study showed the interplay of these three proteins with one another, all impacting the expression from the hTERT minimal core promoter. Although 16E6 could activate the promoter, and it was the only protein that could do so, hTERT promoter-driven expression increases were significantly amplified by overexpression of NFX1-123 and by overexpression of NFX1-123 in its wild-type form with PABPC4 together [27]. Without 16E6, NFX1-123 and PABPC4 could not activate the hTERT promoter-driven expression of luciferase [27]. This exemplified that, although 16E6 could derepress and activate the hTERT promoter, other proteins downstream of transcriptional activation permitted maximal expression from this construct. Several researchers have published foundational studies on the regulation of hTERT and telomerase by 16E6, outside its partnership with NFX1-123 and PABPCs [19,20,40,42,43]; these studies focused on transcriptional regulation of hTERT at its promoter, through cis-elements and the binding or removal of transcription factors. The studies described below provide insight into the partnership of 16E6 with PABPCs and NFX1-123 in regulating hTERT expression at its mRNA level.

## 6. Overexpression of PABPC4 in 16E6 Keratinocytes Showed Increased hTERT mRNA

It was previously found that the PAM2 motif of NFX1-123, which binds to PABPC1 and PABPC4, was critical for NFX1-123 to increase hTERT mRNA and telomerase activity [27]. To further examine the direct role of PABPCs on the endogenous hTERT gene’s mRNA levels and telomerase activity, we quantified the effect of reducing endogenous levels of PABPC1 or PABPC4 in cells and so designed short hairpin RNAs (shRNAs) against specific sequences of PABPC1, PABPC4, or regions common to the two [38]. We first focused on hTERT expression in 16E6-expressing keratinocytes. When both PABPC1 and PABPC4 were reduced by shRNA, there was a decrease in hTERT mRNA levels by approximately half compared with the shRNA scramble control [38]. Additionally, when only PABPC4 was reduced by 99%, hTERT mRNA levels were also cut in half [38]. This highlighted that PABPC4 specifically could impact the levels of hTERT mRNA, despite being expressed at less than 10% of the amount of PABPC1 in cervical cancer cell lines [38].

Although hTERT mRNA levels can be an indirect indicator of telomerase function, we wanted to verify that the decreased levels of hTERT mRNA were functionally relevant to telomerase activity. We employed a telomeric repeat amplification protocol assay to determine the extent of telomerase activity. We found that in 16E6-expressing keratinocytes, with knockdown of PABPC1, PABPC4, or both, there was a significant decrease in telomerase activity [38]. This led us to confirm that when PABPCs were decreased, there was a targeted reduction in hTERT mRNA and a parallel fall in telomerase activity.

Although PABPC1 and PABPC4 were important for optimal hTERT mRNA and telomerase activity in cells with 16E6, we wanted to examine if this required 16E6 co-expression. Therefore, PABPC1 and PABPC4 were knocked down by shRNA in C33A cells, a cervical cancer cell line that does not contain HPV. When PABPC1 or PABPC4 was reduced in the C33A cells, there was no consistent change in hTERT mRNA levels or in telomerase activity [38]. These findings were repeatedly observed in separate experiments, exemplifying that PABPCs influenced hTERT mRNA and telomerase activity only when they were decreased in cells with 16E6.

Lastly, we looked at the effect of PABPC4 overexpression on hTERT in 16E6-expressing keratinocytes, as PABPC4 appeared to be critical to hTERT expression despite its endogenous levels being less than 10% that of PABPC1. 16E6-expressing keratinocytes were transduced with either HA-tagged PABPC4 or an empty vector control; the HA-tagged PABPC4 transduction increased total PABPC4 expression by 150% [38]. In those keratinocytes, hTERT mRNA levels increased by 150%, and telomerase activity increased by nearly 200% [38]. These findings showed that increased PABPC4 in 16E6-expressing keratinocytes increased the hTERT mRNA and telomerase activity [38].

## 7. NFX1-123 Post-Transcriptional Influence in HPV16 E6 Keratinocytes

Because NFX1-123 appeared to be the hub that linked PABPCs, via its PAM2 motif, to the hTERT transcript, and because NFX1-123 has an R3H domain in its unique C-terminus that may directly bind to this transcript, we executed a series of studies to parse the role of NFX1-123 in hTERT post-transcriptional regulation. First, we noted that with deletion of the R3H domain in NFX1-123, overexpression of this mutant form of NFX1-123 did not augment hTERT mRNA or telomerase activity driven by 16E6 in keratinocytes [29]. Second, we found that wild-type NFX1-123 could pull down hTERT mRNA, produced in keratinocytes co-expressing 16E6, in RNA immunoprecipitation assays [29]. Third, we found that in 16E6-expressing keratinocytes with overexpressed NFX1-123, hTERT mRNA stability was improved in RNA decay assays when compared with cells with endogenous levels of NFX1-123 or when compared with an endogenous housekeeping gene’s mRNA [29]. Fourth, when luciferase fused with the 5′ UTR of hTERT was in vitro transcribed, capped, and poly(A) tailed, and then added to whole-cell extracts of keratinocytes with 16E6 and overexpressed NFX1-123, the hTERT/luciferase fused mRNA had a longer half-life in RNA decay assays than in vitro-produced luciferase mRNA fused with the 5′UTR of beta-actin (of similar base pair length) [29]. Together, these studies defined how NFX1-123 regulated hTERT expression with 16E6. The regulation was post-transcriptional through stabilization of the hTERT transcript when NFX1-123 was overexpressed. Two specific components of this regulation were also identified. For the protein component, NFX1-123 required both its PAM2 motif and its R3H domain; when in its full-length, wild-type form, NFX1-123 could bind the hTERT transcript (either directly or in a complex) and recruit PABPCs to increase the hTERT mRNA stability. For the mRNA component, the 5′ UTR sequence of hTERT was required for NFX1-123 to target and increase the stability of that RNA product [29].

Previous microarray analyses of 16E6-expressing keratinocytes indicated that Notch1 mRNA was also increased with higher expression of NFX1-123 [44]. This prompted an investigation into the influence of NFX1-123 both with and without 16E6 on Notch1, a second critical gene in cellular fate and the balance of growth and differentiation in keratinocytes. Similar to what was seen in studies of hTERT, Notch 1 mRNA was increased in 16E6-expressing keratinocytes with overexpressed NFX1-123 [44]. Like hTERT, luciferase assays were conducted to determine whether NFX1-123, with or without 16E6, affected Notch1 promoter-driven luciferase expression and whether the promoter of Notch1 in the luciferase reporter construct also included its 5′ UTR. In keratinocytes, when full-length, wild-type NFX1-123 was transfected with the luciferase reporter construct, there was a two-fold increase in luciferase activity even without 16E6 co-transfection [44]. This denoted that, unlike hTERT, NFX1-123 itself had a direct role in Notch 1 expression even in the absence of 16E6.

However, when 16E6 was co-transfected with NFX1-123, the luciferase activity increased five-fold [44]. This significant augmentation in activity indicated that 16E6 amplified NFX1-123 interaction with Notch1, but it was not required. When the PAM2 motif or the R3H domain was deleted from NFX1-123 in the transfected vector, the augmented luciferase activity fell [44]. Thus, the PAM2 motif and R3H protein domains of NFX1-123 remained important in increasing Notch1 expression. Notch1 and hTERT are the first two genes found to be collaboratively regulated by 16E6, NFX1-123, and PABPCs. They also defined a new mechanism by which 16E6 could affect a host cell gene and pathway—through post-transcriptional gene regulation.

## 8. Clinical Significance and Future Directions

Many of our previous studies have examined 16E6, in collaboration with the cellular proteins NFX1-123 and PABPCs, to deepen our understanding of its regulation of hTERT. However, it is important to expand our scope to understand how narrowly or broadly NFX1-123 and PABPCs affect post-transcriptional gene regulation in the HPV life cycle and in HPV-associated cancer development and progression. Most studies on the E6 protein and its partnership with NFX1-123 have focused on HPV 16. However, it should be noted that NFX1-123 levels are also high in HeLa cells, an HPV 18 positive cervical cancer cell line [45], which one could infer means NFX1-123 is also important in the function of HPV 18 E6. This highlights a potential relationship of NFX1-123 with other HPV types, and additional studies are needed to identify those partnerships. These future studies are important because NFX1-123 is highly expressed in several HPV-associated cancers [46,47,48], because homologs of the *NFX1* gene are found in yeast, plants, and multiple animal species, and because NFX1 functions in metabolism, stress tolerance, and the immune system, and has known functions in cell survival, metabolism, gene expression, and protein modifications [reviewed in 46].

Because NFX1-123 is cytoplasmic and contains domains that allow it to bind to mRNA indirectly through its PAM2 motif and its binding with and recruitment of PABPCs, and directly through its unique R3H domain, future studies will examine other transcripts NFX1-123 may regulate, as directed through 16E6. Similarly, we have seen that PABPCs such as PABPC1 and PABCP4 increase hTERT expression in the presence of 16E6 and wild-type NFX1-123; therefore, we would also like to elucidate if there are additional transcripts governed by PABPCs in the presence of 16E6. All these studies will lead to an even deeper understanding of the role 16E6 plays in post-transcriptional gene regulation.

## 9. Conclusions

**Figure 4:**
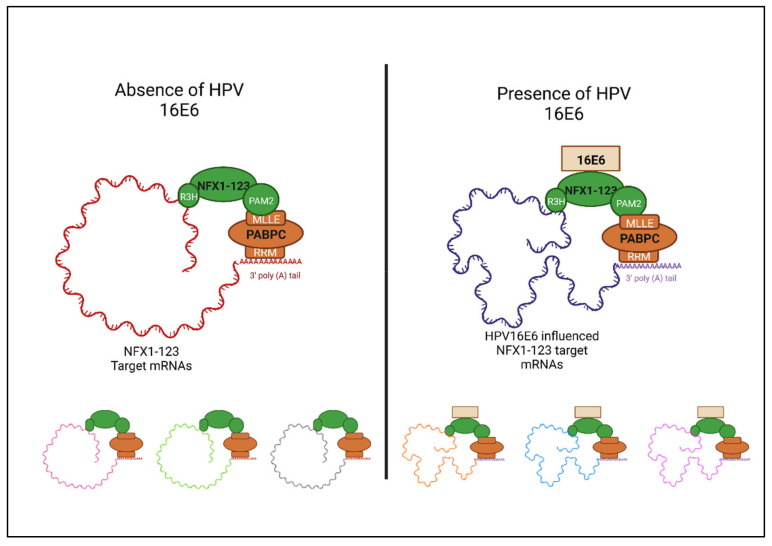
**Schematic of influence of HPV 16E6 in partnership with NFX1-123.** NFX1-123 has the capabilities to bind to mRNA both directly and indirectly. The R3H domain of NFX1-123 contains putative mRNA binding capabilities, which enable NFX1-123 to bind directly to the target transcript. The PAM2 motif of NFX1-123 binds to PABPCs at the MLLE domain. PABPCs can bind to the poly (A) tail of mRNA and help prevent deadenylation of the transcript. The binding to PABPCs allows NFX1-123 to indirectly bind to target mRNAs, which allows for increased stability of the transcripts. In the presence of HPV 16E6, this mRNA signature of NFX1-123 targets shifts. There are different pools of mRNAs that NFX1-123 binds to with or without 16E6, which are represented by the smaller complexes at the bottom of the figure. Figure created with Biorender.com.

As modeled in Figure 4, NFX1-123 and PABPCs normally may interact with one another through their PAM2 and MLLE motifs, respectively, to bind to and regulate mRNAs post-transcriptionally (left panel). However, in the context of 16E6, we also know that NFX1-123 and PABPCs are specifically co-opted to regulate mRNAs that they would otherwise not affect (right panel). This hijacking of normal host proteins by 16E6 to augment the expression of key genes and to fully activate cascades targeting cellular immortalization, longevity, and differentiation is not new. However, the mechanism by which this augmentation and activation occur—post-transcriptional gene regulation—is.
